# Low Distortion of Noise Filter Realization with 6.34 V/μs Fast Slew Rate and 120 mV_p-p_ Output Noise Signal

**DOI:** 10.3390/s21031008

**Published:** 2021-02-02

**Authors:** Fang-Ming Yu, Kun-Cheng Lee, Ko-Wen Jwo, Rong-Seng Chang, Jun-Yi Lin

**Affiliations:** 1 Department of Electrical Engineering/Information and Communication Engineering, St. John’s University, New Taipei City 25135, Taiwan; 2 Department of Optics and Photonics, National Central University, Taoyuan City 32001, Taiwan; s107286004@dop.ncu.edu.tw (K.-C.L.); s103286008@dop.ncu.edu.tw (K.-W.J.); rschang@dop.ncu.edu.tw (R.-S.C.); zivlin@eepv.com.tw (J.-Y.L.)

**Keywords:** low distortion noise filter, Gaussian noise, high order harmonics, fast response, small phase delay

## Abstract

In order to reduce Gaussian noise, this paper proposes a method via taking the average of the upper and lower envelopes generated by capturing the high and low peaks of the input signal. The designed fast response filter has no cut-off frequency, so the high order harmonics of the actual signal remain unchanged. Therefore, it can immediately respond to the changes of input signal and retain the integrity of the actual signal. In addition, it has only a small phase delay. The slew rate, phase delay and frequency response can be confirmed from the simulation results of Multisim 13.0. The filter outlined in this article can retain the high order harmonics of the original signal, achieving a slew rate of 6.34 V/μs and an almost zero phase difference. When using our filter to physically test the input signal with a noise level of 3 Vp-p Gaussian noise, a reduced noise signal of 120 mVp-p is obtained. The noise can be suppressed by up to 4% of the raw signal.

## 1. Introduction

Electronic signals are widely used in our daily lives. In fact, all the information in the environment, such as sounds heard, images seen, and even the electrical signals of the human body, can be converted into electronic signals in different ways for various applications. In general, these signal sources comprise two kinds of signals: the first one is the real signal that we want to obtain, and the second one is the noise signal. Note that the primary physiological signals obtained by biomedical signal measurement systems are mainly low-frequency signals, such as electrocardiography (ECG) measurement, electroencephalography (EEG) measurement, electromyography (EMG) measurement, electroneurography (ENG) measurement [1], etc. Physiological signals obtained by these measurement systems can reflect and aid in diagnosing physical conditions [2,3,4]. With such relevant application to the medical field, the importance of eliminating noise in low-frequency signals is clear. The dedicated work of several medical studies [5,6,7,8] also points toward further work in finding ways to both efficiently and cost-effectively reduce noise. However, in current methods of noise reduction for low-frequency signals, the most common signal processing method is to use a low-pass filter to retain low-frequency information. If the noise in the input signal has a lower frequency than the high order harmonics of the real signal, a general low-pass filter is not sufficient, as only a cut-off frequency lower than the noise can be given. While eliminating the noise, the high order harmonics will also be eliminated, resulting in a low slew rate (SR) and severe signal distortion. On the other hand, when the cut-off frequency is set higher than the noise, it essentially invalidates the filter, so there is the dilemma.

The purpose of this research is to design a noise filter that can eliminate noise and achieve low phase distortion, fast response, and preserve the original signal characteristics. The presented method is effective for noise elimination in cases where the noise meets the criteria of symmetry and possesses random properties. This is a new methodology for noise filtering that uses the characteristics of Gaussian distribution of noise, meaning relevant academic texts are extremely limited or dated. However, this research builds on current methods for analyzing the distribution characteristics of noise [9,10] to design a low distortion noise filter. The simulation results of the general second-, third-, and fourth-order low-pass filters show that there are phase distortions, indicating the inability to retain high order harmonics information [11,12,13]. In our experimental proposal, the simulation is performed by using Multisim 13.0 with a sine wave and square wave as the signal source. The first step is to mix the real signal with the noise. Then we input the mixed signal into the general second-, third-, fourth order low-pass filter and the designed low-distortion noise filter. With the data we gathered, we proceeded to compare the designed low-distortion noise filter with the general second-, third-, and fourth-order low-pass filters, quantizing the attenuation amplitude of the noise, observing the frequency response and analyzing the slew rate to compare the ability of each filter in noise elimination, phase distortion, response speed, and the ability of real signal retention. When compared with use of the average peaks method [14], the presented method can achieve a fast slew rate of 6.34 V/μs and the output noise can be suppressed up to 4%, which is better than the slew rate of 472 V/ms and the 9% suppressed noise of the previous work [14]. This low-distortion filter can directly capture the high and low peak values of the input signal, using RC charge and discharge principle to generate the signal’s upper and lower envelopes and then take their average to reduce the noise. This design can handle noise easily without complex digital signal processor (DSP) processing [15,16,17], and the circuit design is simple and features low power consumption.

## 2. Mathematics Background

**Figure 1:**
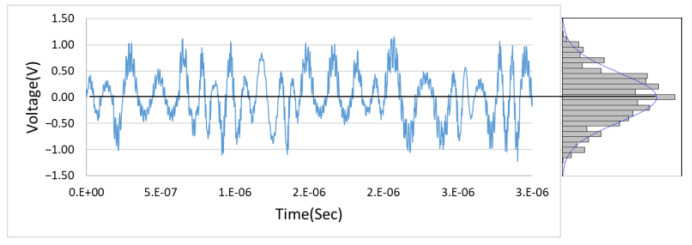
An estimate of Gaussian random variable distribution.

**Figure 2:**
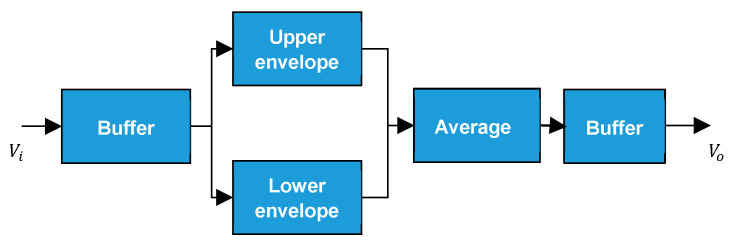
System architecture diagram.

The main purpose of this study is to design a noise filtering circuit. As mentioned in the previous section, thermal noise and shot noise are commonly seen in various types of sensors, and these noise types are considered Gaussian distribution of noise. Figure 1 shows an estimate of Gaussian random variable distribution of the noise, which is generated by Multisim Function Generator and its distribution is similar to Gaussian distribution [12]. However, Gaussian distribution of noise has an important feature, that is, the Gaussian distribution of noise is a noise with random probability distribution, six standard deviations can contain 99.9997% of the entire noise distribution. Therefore, since the signal is random, in this article, we will use the Gaussian distribution characteristics as well as use the RC charge and discharge characteristics to capture the upper and lower envelopes of the input signal with noise and average them to achieve the elimination of the noise. In this research, the overall system architecture, as shown in Figure 2, can be divided into three parts; buffer, capturing of upper and lower envelopes, and the operations of the signal. In Figure 2, the voltage value of the input signal $v_{i}$ is the voltage value of the original signal $v_{s}$ joins with the voltage value of the noise $v_{n}$ and $v_{o}$ is the voltage value of the system output. From Figure 2, it is found that by using the characteristics of Gaussian distribution to capture the high and low envelopes and then averaging them to maintain the integrity of the true signal and to effectively suppress the noise. The upper and lower envelopes are generated by RC charging and discharging circuits.

**Figure 3:**
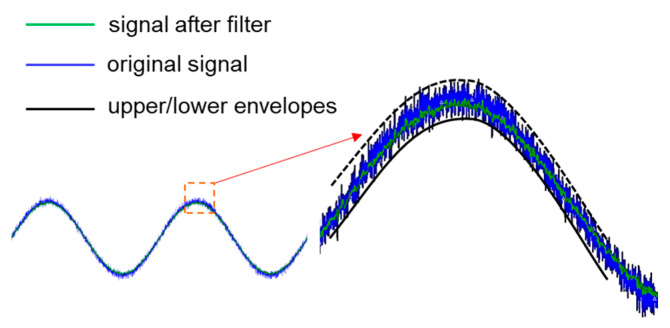
Upper/lower envelopes captured schematic.

**Figure 4:**
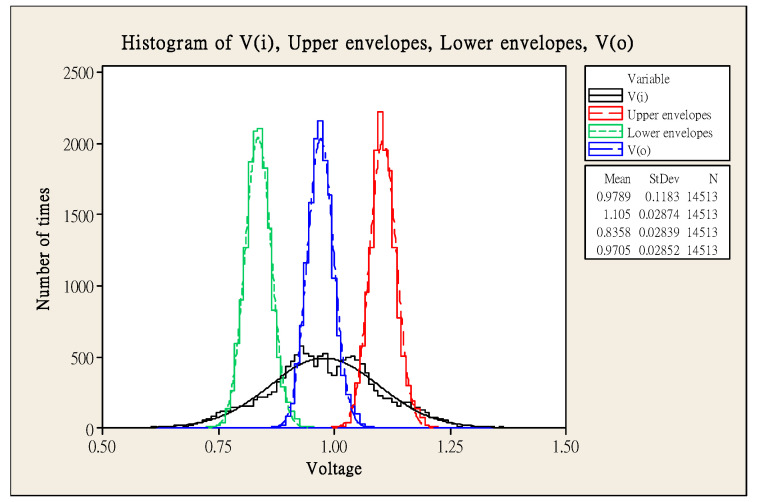
Normal distribution of $v_{i}$, $v_{o}$ and upper/lower envelopes.

An example is given in Figure 3, using a sine wave with noise, the edge signals are captured from the upper and lower edges of the signal for data analysis. As shown in Figure 4, it can be observed that the captured upper and lower envelopes can show a Gaussian distribution, and it is expected that the corresponding peaks can be averaged. This figure shows that the noise standard deviation of the input signal is 0.1183, and the captured upper and lower envelopes can reduce the standard deviation to 0.028, which is about 23% of the raw signal, proving that this method can indeed attenuate the noise.

## 3. Design Method

### 3.1. Circuit Design: Retention of the Real Signal

**Figure 5:**
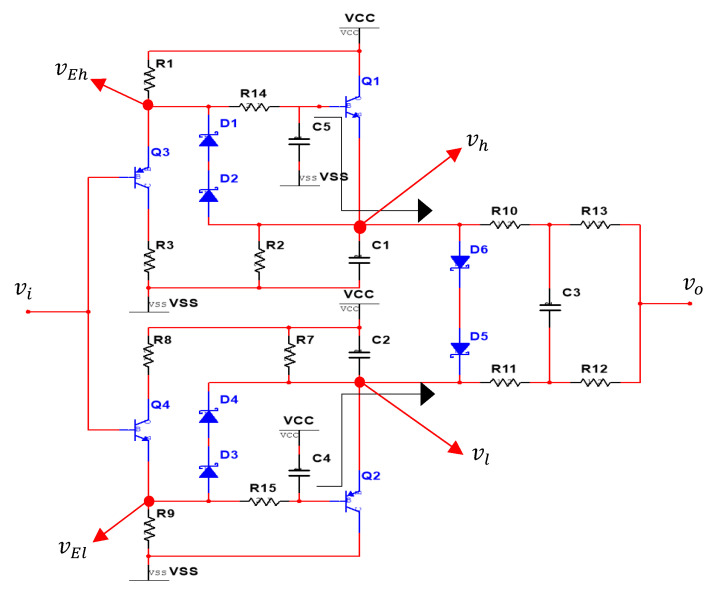
The designed noise filter (named BJT) circuit.

In circuit design, the transistor is used as a buffer, and the RC loop formed by resistor and capacitor is used to capture the upper and lower envelopes information. Finally, a voltage divider circuit formed by a resistor is used for averaging the upper and lower envelope signals. The overall designed noise filter (named BJT) circuit is proposed as shown in Figure 5.

In Figure 5, the signal is transmitted from the input to the output. Since it does not need too much attenuation, two transistors of PNP and NPN forms are set. When the signal is transmitted to the first stage of the transistors (as a buffer) Q_3_ and Q_4_, the signal is transmitted from the base to the emitter. There will be a superposition of the forward voltage $v_{be}$, so the signal of the upper and lower envelopes can be listed as:

(1) $$\mathrm{Upper}\;\mathrm{envelope}:\;v_{Eh}=v_{i}+v_{be3}$$

(2) $$\mathrm{Lower}\;\mathrm{envelope}:\;v_{El}=v_{i}-v_{be4}$$

where $v_{i}=v_{s}+v_{n}$; $v_{s}$ is the voltage value of the original signal, and $v_{n}$ is the voltage value of the noise.

Then the signal of $v_{i}$ is transmitted to the second-stage of transistors Q_1_ and Q_2_. In addition to being a buffer, the function here is to eliminate the forward bias superposed by the first-stage transistors. By the upper RC circuit, the upper envelope of signal $v_{h}$ will be $\left(v_{s}+v_{nh}+v_{be3}\right)-v_{be1}$; $v_{nh}$ is the upper envelope of $v_{n}$. And by the lower RC circuit, the lower envelope of signal $v_{l}$ will be $\left(v_{s}-v_{nl}-v_{be4}\right)+v_{be2}$; $v_{nl}$ is the lower envelope of $v_{n}$. Normally, $v_{nh}$ is equal to $v_{nl}\;(v_{nh}\approx v_{nl})$. Therefore, the upper envelope of $v_{h}$ and lower envelope of $v_{l}$ can be expressed as follows:

(3) $$\mathrm{Upper}\;\mathrm{envelope}:\;v_{h}=v_{s}+v_{nh}+v_{be3}-v_{be1}$$

(4) $$\mathrm{Lower}\;\mathrm{envelope}:\;v_{l}=v_{s}-v_{nl}-v_{be4}+v_{be2}$$

In Equations (1)–(4), the $v_{be1}$, $v_{be2}$, $v_{be3}$ and $v_{be4}$ are the forward bias between the base and emitter of Q_1_, Q_2_, Q_3_ and Q_4_, normally influenced by the temperature (T), $V_{\mathrm{cc}}$ and $V_{\mathrm{ss}}$, we can name as $v_{be1}\left(T,V_{\mathrm{cc}},V_{\mathrm{ss}}\right)$, $v_{be2}\left(T,V_{\mathrm{cc}},V_{\mathrm{ss}}\right)$, $v_{be3}\left(T,V_{\mathrm{cc}},V_{\mathrm{ss}}\right)$ and $v_{be4}\left(T,V_{\mathrm{cc}},V_{\mathrm{ss}}\right)$. The voltage value of $v_{be}$ can be expressed as follows [18]:

(5) $$\begin{array}{c} v_{be}=\frac{kT}{q}ln\frac{I_{c}}{I_{s}}\\ =\frac{1.3805\times 10^{-23}JK^{-1}}{1.602\times 10^{-19}C}ln\frac{I_{c}}{I_{s}} \end{array}$$

where *J* is energy (Joule), *K* is absolute temperature, *C* is unit of charge (Coulombs), $I_{c}$ is the collector current (mA) and $I_{s}$ is the reverse saturation current (pA).

In theory, the forward bias of each transistor will be the same, but there are still slight differences in practical components, and the voltage drift between components will also occur depending on the operating temperature. Nevertheless, the main purpose of the filter design is to eliminate the noise. To avoid the voltage drift problems, we chose PNP & NPN packaged in one group of two components carriers, Q_3_ and Q_1_ are in one group, Q_2_ and Q_4_ are another group.

In this way, we can see that $v_{be3}$ minus $v_{be1}\;(v_{be3}-v_{be1})$, and negative $v_{be4}$ plus $v_{be2}$ $\left(-v_{be4}+v_{be2}\right)$ can approach zero, and the temperature drift voltage can be avoided due to the different temperature of each component. Thus, the final expression of the output voltage can be listed as:

(6) $$\begin{array}{c} v_{o}=\frac{v_{h}+v_{l}}{2}\\ =\frac{v_{s}+v_{nh}+v_{be3}-\;v_{be1}+v_{s}-v_{nl}-v_{be4}\;+v_{be2}}{2}\\ =\frac{v_{s}+v_{s}}{2}=v_{s} \end{array}$$

Therefore, from Equation (6), it is demonstrated that the average of the upper and lower envelopes can achieve the elimination of noise and the retention of the real signal.

### 3.2. Circuit Design: Capturing of Upper and Lower Envelopes

**Figure 6:**
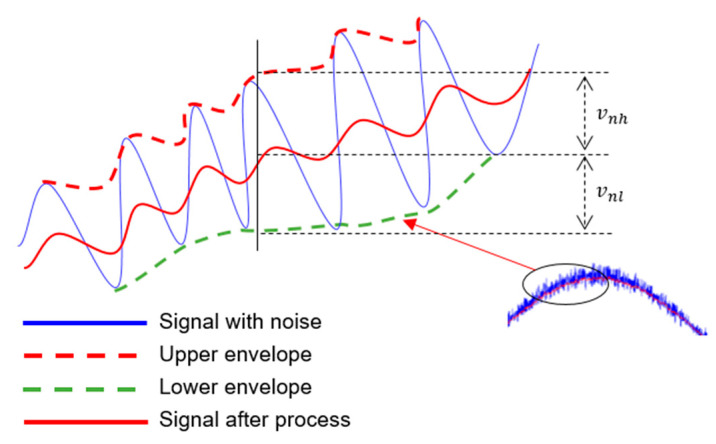
Sinusoidal wave with noise and the Upper/Lower envelopes. ($v_{nh}$ is the upper envelope of $v_{n}$; $v_{nl}$ is the lower envelope of $v_{n}$; $v_{n}$ is the noise signal).

As shown in Figure 6, the main method of the circuit design for noise attenuation is to use the high and low peaks of the noise, then by charging and discharging the capacitors and resistors of R_14_, R_15_, C_4_, and C_5_ to capture the high and low envelopes, but this requires special attention to the matching of resistance and capacitance.

**Figure 7:**
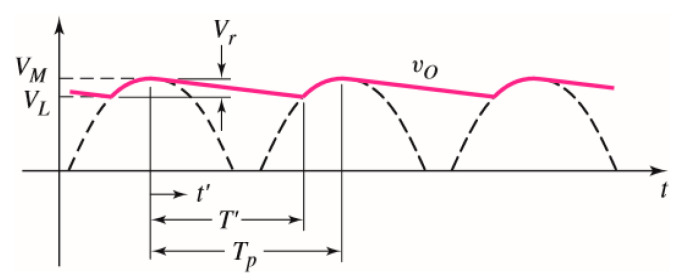
RC filter showing the ripple voltage.

Figure 7 [7] shows the ripple voltage generated by a full-wave rectifier with smoothing capacitor. As shown in Figure 7, the value of RC not only affects the charge and discharge time, but also affects the smoothness of the captured envelope. In fact the higher the value of RC, the better the smoothing. In Figure 7, the time constant of τ and output voltage of $v_{o}$ can be expressed as following:

(7) τ=RC

(8) vo(t)=VMe−t′τ=VMe−t′RC 

In Figure 7, some symbols are defined as follows:

- *V_M_* is the maximum output voltage and *V_L_* is the minimum output voltage.
- *V_r_* is the ripple voltage.
- $t^{\prime }$ is the time for discharging and $T^{\prime }$ is the discharging period.
- *T_p_* is the period of the ripple.

### 3.3. Circuit Design: Fast Charge and Discharge Design

**Figure 8:**
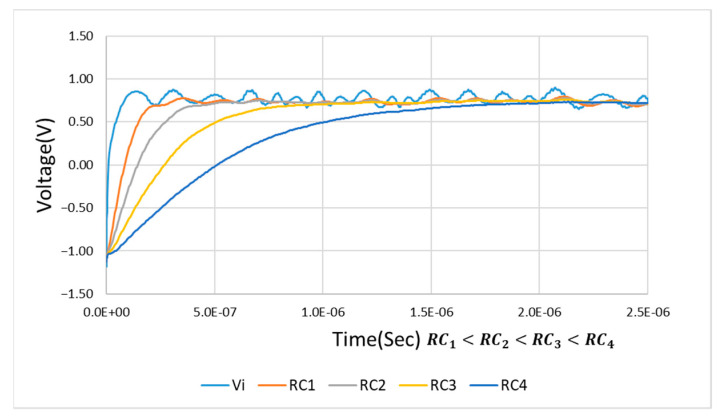
Transient response analysis of charging time.

**Figure 9:**
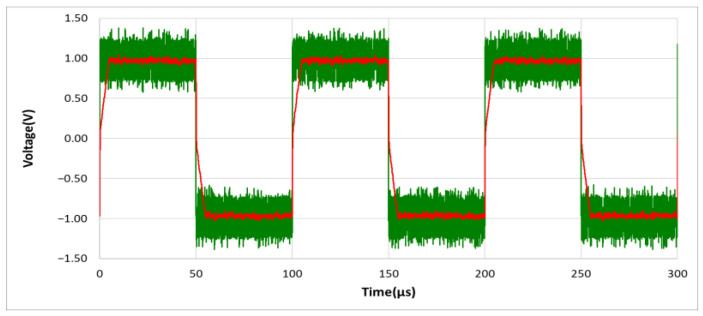
The output waveform distortion of the designed noise filter for only using the RC charging and discharging circuit, without using D_1_–D_6_ Schottky diodes. ($v_{o}$: red line; $v_{i}$: green line).

As shown in Figure 8, the larger the value of time constant τ, the longer the charge and discharge time will be, and the captured envelope will thus be smooth. The opposite occurs under a smaller τ value. If the value of τ is increased, during the rapid changes of the input signal, the distortion will be caused by insufficient charge and discharge time. Figure 9 shows the output of the designed circuit caused by only using RC charging and discharging circuit under the input signal of 10 KHz, 1.5 Vp square wave mixed with 5 Vp-p noise. The noise is generated by the Multisim Function Generator Interface.

**Figure 10:**
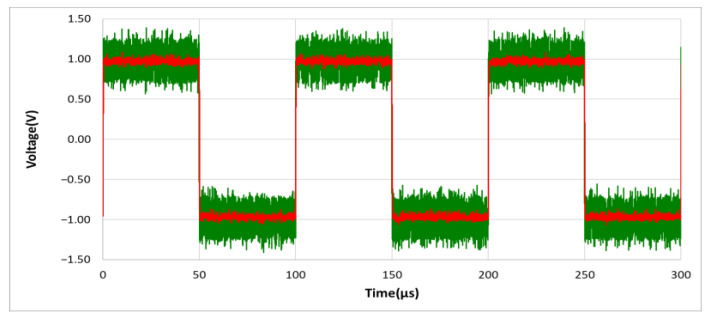
The output waveform distortion is improved using the designed noise filter circuit, with RC circuit and D_1_–D_6_ Schottky diodes. ($v_{o}$: red line; $v_{i}$: green line).

**Figure 11:**
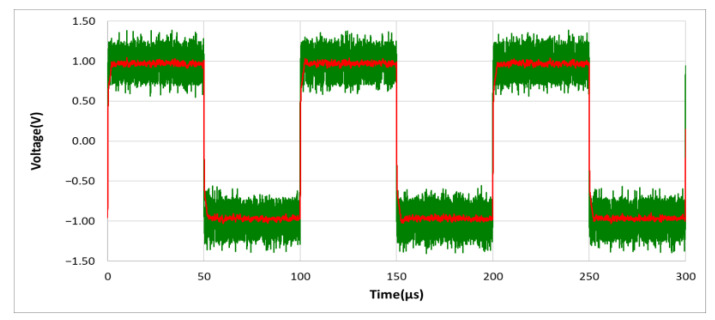
The output waveform of the designed noise filter, without D_5_ and D_6_ Schottky diodes. ($v_{o}$: red line; $v_{i}$: green line).

As shown in Figure 9, the output waveform distortion of the designed noise filter is caused when only using the RC charging and discharging circuit without using D_1_–D_6_ Schottky diodes. In order to achieve high noise removal ability and fast response speed as well as keep the signal undistorted, in the designed noise filter of Figure 5, we set the Schottky diodes between the base and emitter of Q_1_ and Q_2_, and between $v_{l}$ and $v_{h}$, using their forward bias conduction characteristics as a fast discharge circuit switch. In other words, when the instantaneous change of the voltage value of the input signal $v_{i}$ $\left(\Delta v_{i}\right)$ is greater than the voltage value of the diode forward bias of $V_{D}$ $\left(\Delta v_{i}>V_{D}\right)$, a fast discharge is performed. It’s important to note that the Schottky diode is used in our design, mainly because of its characteristics of fast switching and low forward bias voltage. In general, the forward bias of a typical diode is about 0.7~1.7 volts, while the forward bias of a Schottky diode is about 0.15 to 0.45 volts. In our circuit design, we use two Schottky diodes connected in series, so that when $\left(\Delta v_{i}>0.4\;V\right)$, fast discharge is performed. Using the designed noise filter and given the same input signal of 10 KHz, 1.5 Vp square wave mixed with 5 Vp-p noise as in Figure 9, the output result can show that the waveform distortion caused by RC charging and discharging is no longer visible as shown in Figure 10. However, if without using D_5_ and D_6_ Schottky diodes, the output result is shown in Figure 11.

## 4. Experiment Results

### 4.1. Circuit Simulations

The filtering effect of the designed noise filter circuit is verified by Multisim 13.0 software simulation. The verified items include circuit frequency response, phase response, noise filtering capability, response speed, and Fourier amplitude spectrum. In circuit simulations, the cut-off frequency $f_{c}$ of 20 KHz will be used as the verification control group. Meanwhile, the verification method uses an input signal obtained from the pre-stage mixing circuit of an original signal of 10 KHz, 1.5 Vp square wave mixed with 5 Vp-p noise generated by Multisim Function Generator Interface.

#### 4.1.1. Low-Pass Filter

**Figure 12:**
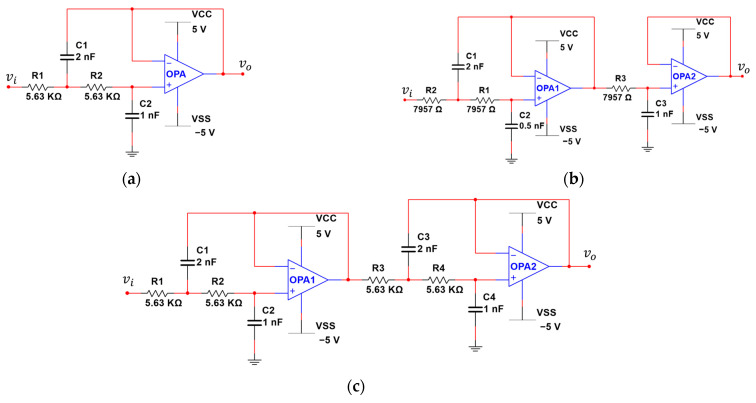
(**a**) Second-order low pass Butterworth filter; (**b**) Third-order low pass Butterworth filter; (**c**) Fourth-order low-pass Butterworth filter.

In this section, the traditional low-pass filters will be introduced. The circuits of second-, third-, and fourth-order low-pass Butterworth filters are shown in Figure 12.

Figure 12a is the RC network of the second-order low-pass filter, composed of two groups of $R_{1}$, $C_{1}$, and $R_{2}$, $C_{2}$, and the cut-off frequency $f_{c}$ in this filter can be expressed as follows:

(9) $$f_{c}=\frac{1}{2\pi \times \sqrt{R_{1}C_{1}R_{2}C_{2}}}$$

Figure 12b shows a third-order low pass filter. This filter is composed of a second-order filter and a first-order filter connected in series, the cut-off frequency $f_{c}$ can be expressed as:

(10) $$f_{c}=\frac{1}{2\pi \times \sqrt[{3}]{R_{1}R_{2}R_{3}C_{1}C_{2}C_{3}}}$$

Figure 12c shows a fourth-order low-pass filter. This filter is composed of two second-order filters connected in series, the cut-off frequency $f_{c}$ can be expressed as:

(11) $$f_{c}=\frac{1}{2\pi \times \sqrt[{4}]{R_{1}R_{2}R_{3}R_{4}C_{1}C_{2}C_{3}C_{4}}}$$

**Figure 13:**
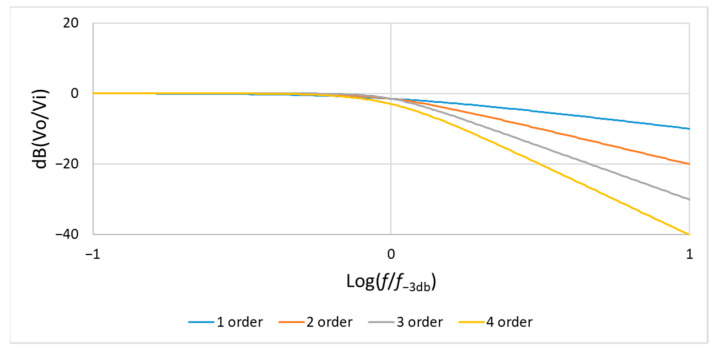
Frequency response of the second-, third-, and fourth-order low-pass Butterworth filters ($f_{-3\mathrm{db}}$ = $f_{c}$).

Figure 13 shows the frequency response of the second-, third-, and fourth-order low-pass Butterworth filters.

In general, for an *n*-th order Butterworth low-pass filter, the gain G(ω) can be expressed as:

(12) $$G^{2}\left(\omega \right)=\frac{G_{0}{}^{2}}{1+{\left(\frac{\omega }{\omega_{c}}\right)}^{2n}}=\frac{G_{0}{}^{2}}{1+{\left(\frac{f}{f_{c}}\right)}^{2n}}$$

where

- *n* = the order of the filter.
- ω = the radian frequency which is equal to 2πf.
- $\omega_{c}$ = cut off radian frequency.
- $f_{c}$ = cutoff frequency = frequency at which the gain drops to −3 dB.
- $G_{0}$ is the DC gain (zero frequency gain).

In the low-pass Butterworth filters of Figure 12, the operational amplifier is connected as a voltage follower, so at zero frequency, its gain is 1, that is $G_{0}=1$ and the frequency response will be attenuated to 0dB. Therefore, if $f=10f_{c}$, then Equation (12) can be expressed as follows:

(13) $$\begin{array}{ll} G^{2}\left(w\right) & =\frac{G_{0}{}^{2}}{1+{\left(\frac{\omega }{\omega_{c}}\right)}^{2n}}=\frac{G_{0}{}^{2}}{1+{\left(\frac{f}{f_{c}}\right)}^{2n}}\\ & ≅\frac{1}{{\left(\frac{f}{f_{c}}\right)}^{2n}}={\left(\frac{f}{f_{c}}\right)}^{-2n}=10^{-2n}={\left(10^{-n}\right)}^{2} \end{array}$$

In this case, |G(ω)| will be $10^{-n}$, that is $\left|G\left(\omega \right)\right|≅10^{-n}$, and the frequency response at $f=10f_{c}$ can expressed as follows:

(14) $$20log\left|G\left(\omega \right)\right|=20log10^{-n}=-20\times n\;\mathrm{dB}$$

As Figure 13 indicates, the slope of the frequency response diagram can be described as −20 × *n* dB/decade when the frequency exceeds $f_{c}$. In Figure 13, we can find once the frequency exceeds $f_{c}$, the slopes of second-, third-, and fourth-order low-pass Butterworth filters are −40 dB, −60 dB, and −80 dB respectively.

#### 4.1.2. Frequency Response Analysis

**Figure 14:**
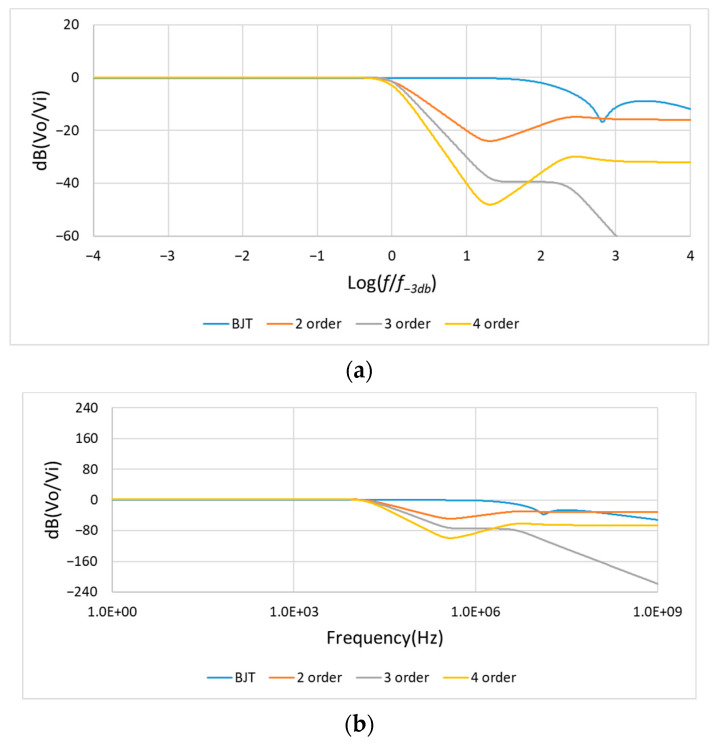
(**a**) Frequency response at *f* = 10$f_{c}$: $\left(\mathrm{Log}\left(\frac{f}{f_{-3\mathrm{db}}}\right)=1\right)$; (**b**) Frequency response from 1 Hz to 1 GHz. (BJT is the designed noise filter, $f_{c}=f_{-3\mathrm{db}}=20\;\mathrm{KHz}$).

As shown in Figure 14a, the traditional second-, third-, and fourth-order low-pass filters have reduced the gain to be −40~−80 dB at the frequency of 10 times of $f_{c}$. However, in Figure 14a, we can see the BJT designed noise filter has a wider frequency response range. Figure 14 and Table 1 show the frequency response of second-, third-, fourth-order, and the BJT designed noise filter. Unlike general filters of cut-off frequency limitation, the proposed noise filter can operate at a wider frequency response range normally within 1 MHz.

#### 4.1.3. Square and Sine Wave Response

**Figure 15:**
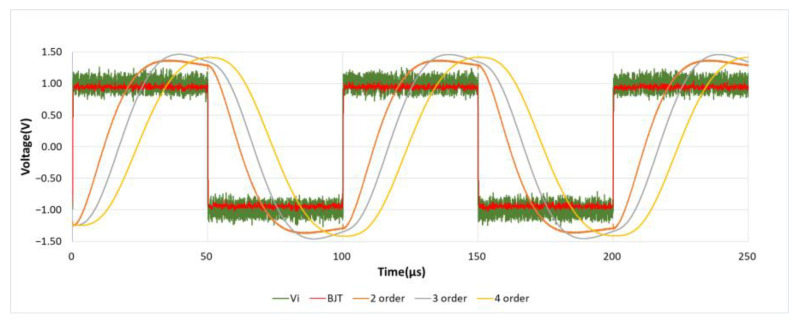
Square wave response.

**Figure 16:**
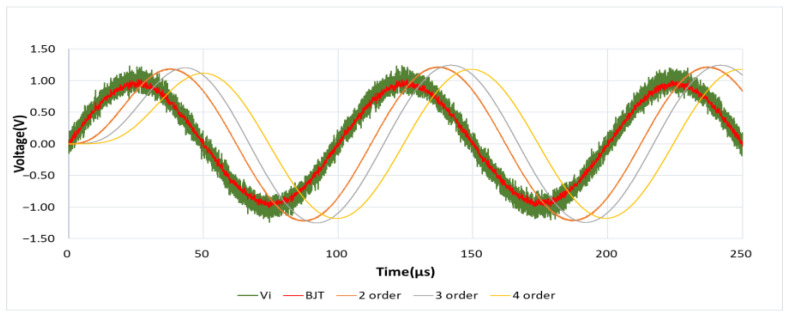
Sinusoidal wave response.

As shown in Figure 15, similarly, a 10 KHz, 1.5 Vp square wave mixed with 5 Vp-p noise generated by Multisim Function Generator is used as the input signal. In unit scale of Time/Div = 50 μs, it can be observed that the performance of this designed circuit has a fast response speed, and there is no waveform distortion caused by the RC charging and discharging time. At the same time, in the simulation results of Figure 16, it can also be observed that there is no sine wave phase shift caused by the delay of RC charging and discharging time when using the BJT noise filter.

#### 4.1.4. Noise Attenuation Analysis

**Figure 17:**
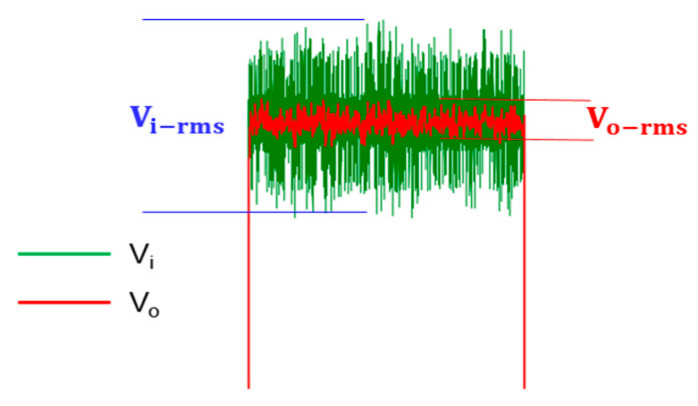
Designed circuit noise filtering behavior.

As described in Section 4.1.3, using the same input signal of 10 KHz, 1.5 Vp square wave mixed with 5 Vp-p noise, the 5 Vp-p noise is generated by Multisim Function Generator Interface. Figure 17 and Table 2 show the designed circuit noise filtering behavior and effect. In Table 2, the one standard deviation (one sigma; 1σ) of the input noise signal is 81 mVrms. It can obtain 19 mVrms of output noise signal. This can show that the amplitude of the noise in one sigma (1σ) is attenuated by 76.5%. Because the noise distribution is a Gaussian distribution, a range of 99.97% noise amplitude can be covered with six standard deviations (six sigma; 6σ). In Figure 17 and Table 2, the six standard deviations (six sigma; 6σ) of the input noise signal is 0.486 Vrms, resulted in the output noise signal of 0.114 Vrms. It shows that the overall noise amplitude is also attenuated by 76.5%.

#### 4.1.5. Response Speed Analysis

**Figure 18:**
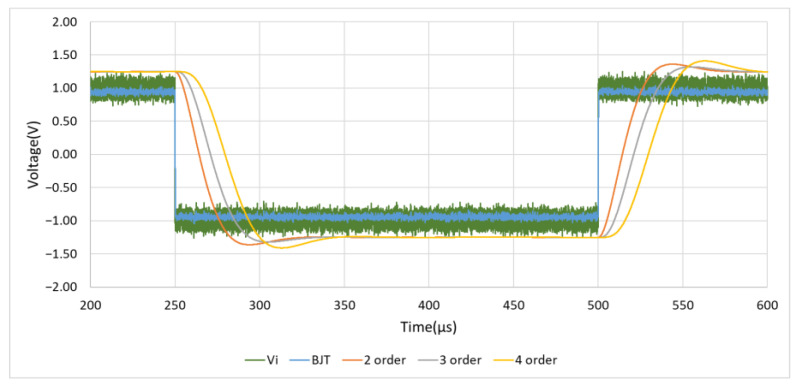
Response speed of the designed noise filter.

As shown in Figure 18 and Table 3, the fastest response speed of this designed noise filter can reach 0.27 μs, it is the time from 10% Vp-p rise to 90% Vp-p. If the voltage variation is taken into consideration, the fast slew rate of 6.34 volts per microsecond (6.34 V/μs) can be obtained, that is the slope of the voltage change in 0.27 μs, which is much greater than the 0.07 volts per microsecond (0.07 V/μs) of the conventional filter. In Table 3, the $\Delta T_{up}$ and $\Delta T_{Down}$ are defined as follows:

1. $\Delta T_{up}$ = Time from 10% Vp-p rise to 90% Vp-p.
2. $\Delta T_{Down}$ = Time from 90% Vp-p fall to 10% Vp-p.

#### 4.1.6. Fourier Amplitude Spectrum Analysis

**Figure 19:**
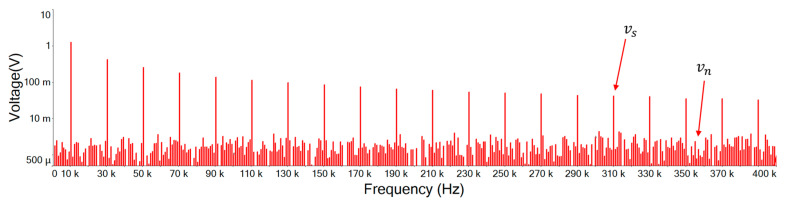
Input signal Fourier amplitude spectrum. ($v_{s}$ is the original signal; $v_{n}$ is the noise signal).

Figure 19 illustrates the Fourier amplitude spectrum of 10 KHz, 1.5 Vp square wave mixed with 5 Vp-p noise of input signal. It can be seen that the noise intensity is similar and scattered in each spectrum. However, as the waveform of the main signal is a square wave, only odd harmonics exist in the frequency space. A Fourier series can be used to represent an ideal square wave, this Fourier series will be represented by an infinite polynomial as follows [19]:

(15) $$\begin{array}{l} x_{square}\left(t\right)=\frac{4}{\pi }\overset{\infty }{\underset{k=1}{\sum }}\frac{sin\left(\left(2k-1\right)2\pi ft\right)}{\left(2k-1\right)}\\ =\frac{4}{\pi }\left(sin\left(2\pi ft\right)+\frac{1}{3}sin\left(6\pi ft\right)+\frac{1}{5}sin\left(10\pi ft\right)+\ldots \right) \end{array}$$

**Figure 20:**
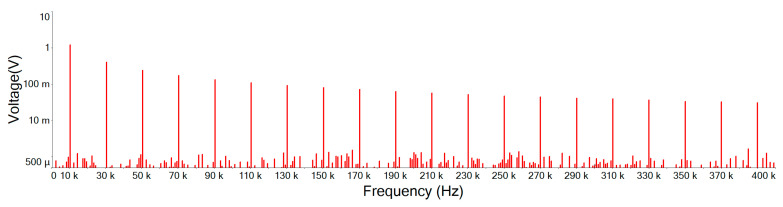
Designed noise filter output signal Fourier amplitude spectrum.

**Figure 21:**
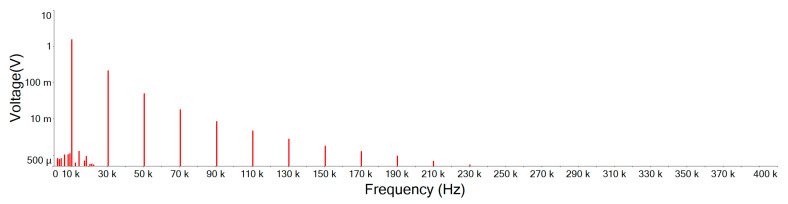
Second-order low-pass filter output signal Fourier amplitude spectrum.

**Figure 22:**
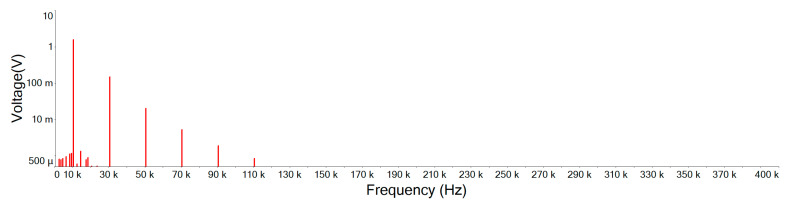
Third-order low-pass filter output signal Fourier amplitude spectrum.

**Figure 23:**
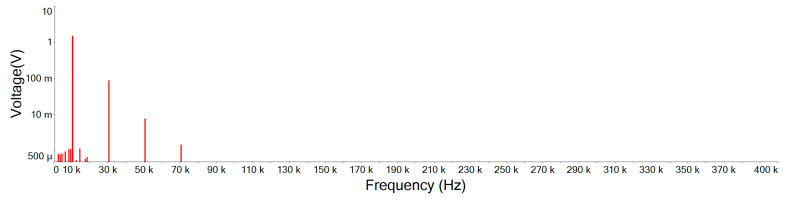
Fourth-order low-pass filter output signal Fourier amplitude spectrum.

Figure 20 is the output signal Fourier amplitude spectrum of the designed noise filter after inputting the input signal of Figure 19. It shows that the noise is attenuated and the high order harmonics of the main signal still remain. Comparing this designed noise filter with traditional filters of Figure 21, Figure 22 and Figure 23, where the noise below the cut-off frequency of $f_{c}$ still remains, but the high order harmonics of the main signal of exceeding frequency $f_{c}$ are obviously attenuated. In Figure 20, and Table 4, we can see that the designed noise filter maintains the integrity of the true signal and effectively suppresses the noise. In addition, in Table 4, we can find if the quintuple harmonics Fourier amplitude spectrum peak value of the input signal is 0.25305 V, after this designed filter, the output Fourier amplitude spectrum peak value of the quintuple harmonics is 0.24055 V. In this case of traditional second-, third-, and fourth-order low-pass filters, the output Fourier amplitude spectrum peak value of the quintuple harmonics has decayed to be 0.049239 V, 0.019903 V, and 0.007709 V.

#### 4.1.7. Phase Response Analysis

**Figure 24:**
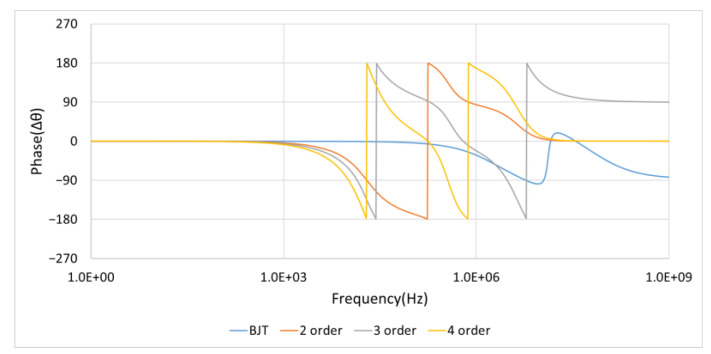
Phase response of second-, third-, fourth-order, and BJT filter. (BJT is the designed noise filter, $f_{c}=f_{-3dB}=20\;\mathrm{KHz}$).

Figure 24 and Table 5 illustrate the phase response and the lead or lag angle values in a 10 KHz, 1.5 Vp square wave mixed with 5 Vp-p noise of input signal. Thanks to the utilization of the RC circuit accompanied with the Schottky diodes in this filter for capturing the upper envelope and the lower envelope, the amount of stored charge is extremely small, which results in the extremely small phase delay. In Table 5, we can see the phase delay of the proposed noise filter has little lag and less than 3.43 degrees within 100 KHz. As for the other filters, the phase shift is much larger than this value.

### 4.2. Circuit Implementation

**Figure 25:**
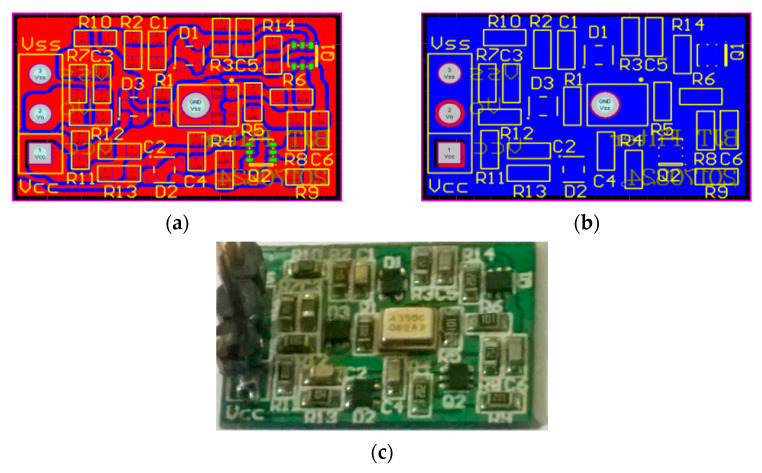
(**a**) Component side of the designed noise filter PCB Layout; (**b**) Soldering side of the designed noise filter PCB Layout; (**c**) The designed noise filter product.

Figure 25 shows the designed noise filter PCB layout and the final product. The PCB layout design is developed by Protel 99 SE. The size of the noise filter is 12 mm × 19 mm with a cost of less than NT$100. Using this method of noise filtering, the input signal can be directly processed through the circuits without performing complicated operations such as DSP.

**Figure 26:**
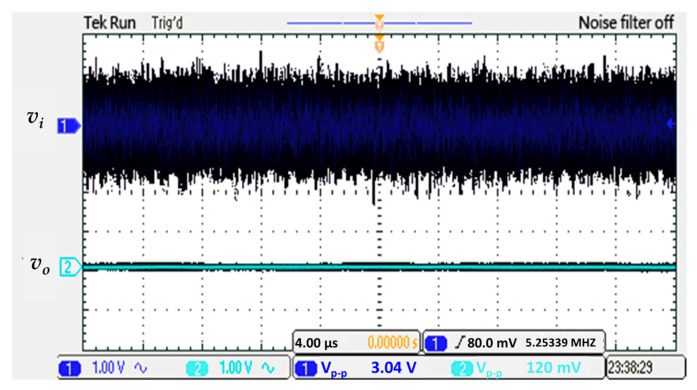
Test of noise filtering effect on real circuit board.

Figure 26 shows the input signal with noise of 3.04 Vp-p, after the designed noise filter of circuit implementation test, a 120 mVp-p output noise signal can be obtained, which can reduce the input noise up to 4% of itself.

## 5. Conclusions

### 5.1. Technique Features

Signal processing and noise filtering have always been very important subjects in technology, whether used in measurement or communication. This research investigates the distribution characteristics of the noise commonly found in electronic circuits, and applies a scientific understanding of the development of noise filter technology. According to the noise distribution characteristics, this paper proposes a method to split the signal source into two parts, separately extracting the high peaks and the low peaks by individual circuit design, and using the RC charge and discharge principle to form the upper envelope signal ($v_{h})$ and the lower envelope signal ($v_{l})$. Further, the extracted upper and lower envelopes and the input signal all have a Gaussian distribution. Therefore, by taking the upper envelope generated by the original signal plus noise signal and the lower envelope generated by the original signal minus noise signal, the numbers are averaged to reduce the Gaussian noise. Significantly, the noise filter of this design has no cut-off frequency, so the high order harmonics of the actual signal remain unchanged until 1 MHz. This is limited by the operation speed of the transistor. Thanks to the fast response of the noise filter, the input signal can be kept up to 6.34 V/μs and the actual signal integrity is preserved, which is better than the previous work [14] in a slew rate of 472 V/ms. Also, by utilizing the RC circuit accompanied with Schottky diodes in the circuit design, a much smaller phase delay is achieved than when a conventional filter is used. Several important features of the proposed technique are summarized as following:

● High response speed

● At the same input frequency signal, unlike a traditional filter, this filter can be operated at a slew rate (charge and discharge slope) of 6.34 V/μs. Under the same conditions, the traditional filter can only reach at a speed of 0.09 V/μs.

● Minimal phase delay

A traditional filter is delayed due to the charging of the RC circuit. However, the circuit in the filter designed here creates a high response speed through the combined use of the RC charging and discharging circuit and the Schottky diodes. Since the above circuits capture the upper envelope and the lower envelope, the quantity of stored electric charge is extremely small, and the resulting phase delay is correspondingly small. The phase delay is approximately 3 degrees.

● Noise attenuation

In the circuit simulation test, an input noise signal of 0.486 Vrms is given to this filter, achieving an output noise signal of 0.11 Vrms, with an attenuation of 25% of the raw signal. Through the physical PCB circuit test, an input signal with 3 Vp-p Gaussian Noise, after this noise filter, achieves the output noise signal of 120 mVp-p. The noise can be suppressed to 4% of the raw signal.

● Maintain signal integrity

Rather than following the traditional filter, the range of the passband is set at the cutoff frequency, but this filter is not affected by the cutoff frequency. After the signal is transmitted from the filter, the high order harmonics information within 1 MHz can still be retained. The Fourier amplitude spectrum peak values shown in Table 4 reveal that the quintuple harmonic Fourier amplitude spectrum of the raw signal is 0.25 V, and the quintuple harmonic output from the designed filter is 0.24 V. Compared with the traditional second-order low-pass filter, the quintuple harmonic output signal has decayed to 0.049 V, while in the fourth-order low-pass filter, the output signal of quintuple harmonic is decayed even more to 0.0077 V.

### 5.2. Future Prospects

The most important aspect of creating a noise filter is to match a detection system or a measurement system to assist the noise filtering, as to highlight its value. In the future, it is hoped that this structure can be used in measurement systems to assist in noise removal.

Although the noise filter in this study can attenuate the Gaussian distributed noise, it is limited by the frequency response of using the transistors in the overall filters, the output signal gain will begin to attenuate at about 1 MHz when using transistor components. However, if the noise filter is applied to signals around 200 kHz, in square wave, 5 times of this frequency of high order harmonics will fall at the position of 1 MHz, in this case it will cause distortion. Addressing this distortion would be a valuable direction for future research to take.

## Figures and Tables

**Table 1:** The dB values of frequency response at 1 KHz, 10 KHz, 200 KHz, and 1 MHz. ($f_{c}=f_{-3\mathrm{db}}=20\;\mathrm{KHz}$).

Frequency	BJT	2 Order	3 Order	4 Order
1 KHz	0 dB	0 dB	0 dB	0 dB
10 KHz	0 dB	0 dB	0 dB	0 dB
200 KHz	0 dB	−40 dB	−60 dB	−80 dB
1 MHz	−1 dB	−42 dB	−78 dB	−84 dB

**Table 2:** Designed circuit noise filtering effect.

	1σ	3σ	6σ
$V_{i-\mathrm{rms}}$	0.081 V	0.241 V	0.486 V
$V_{o-\mathrm{rms}}$	0.019 V	0.057 V	0.114 V

**Table 3:** The data of the designed noise filter response speed.

Items	Δ*T*_*up*_ (μs)	Δ*T*_*Down*_ (μs)
BJT	0.27	0.27
2 order	25.48	25.47
3 order	31.57	31.53
4 order	33.69	33.63

**Table 4:** Fourier amplitude spectrum peak values. (BJT is the designed noise filter).

Frequency	$v_{i}$	BJT	2 Order	3 Order	4 Order
10 KHz	1.25896	1.20108	1.54432	1.58431	1.5003
30 KHz	0.41925	0.401337	0.212284	0.148126	0.085771
50 KHz	0.25305	0.24055	0.049239	0.019903	0.007709
70 KHz	0.180795	0.171364	0.017986	0.005179	0.001454

**Table 5:** The lead or lag angle values of phase response at 1 KHz, 10 KHz, 100 KHz, and 1 MHz. ($f_{c}=f_{-3dB}=20\;\mathrm{KHz}$).

Frequency	BJT	2 Order	3 Order	4 Order
1 KHz	−0.04°	−5.09°	−7.63°	−10.16°
10 KHz	−0.36°	−55.77°	−79.56°	−111.17°
100 KHz	−3.43°	−170.14°	110.90°	19.75°
1 MHz	−34.36°	83.59°	−22.37°	166.83°

## Data Availability

Not applicable.
